# Genomic Prediction Using Multi-trait Weighted GBLUP Accounting for Heterogeneous Variances and Covariances Across the Genome

**DOI:** 10.1534/g3.118.200673

**Published:** 2018-09-07

**Authors:** Emre Karaman, Mogens S. Lund, Mahlet T. Anche, Luc Janss, Guosheng Su

**Affiliations:** Center for Quantitative Genetics and Genomics, Aarhus University, 8830 Tjele, Denmark

**Keywords:** Genomic prediction, GenPred, Shared Data Resources, Genetic architecture, Genomic relationship matrix, Region size, SNP weight

## Abstract

Implicit assumption of common (co)variance for all loci in multi-trait Genomic Best Linear Unbiased Prediction (GBLUP) results in a genomic relationship matrix (**G**) that is common to all traits. When this assumption is violated, Bayesian whole genome regression methods may be superior to GBLUP by accounting for unequal (co)variance for all loci or genome regions. This study aimed to develop a strategy to improve the accuracy of GBLUP for multi-trait genomic prediction, using (co)variance estimates of SNP effects from Bayesian whole genome regression methods. Five generations (G1-G5, test populations) of genotype data were available by simulations based on data of 2,200 Danish Holstein cows (G0, reference population). Two correlated traits with heritabilities of 0.1 or 0.4, and a genetic correlation of 0.45 were generated. First, SNP effects and breeding values were estimated using BayesAS method, assuming (co)variance was the same for SNPs within a genome region, and different between regions. Region size was set as one SNP, 100 SNPs, a whole chromosome or whole genome. Second, posterior (co)variances of SNP effects were used to weight SNPs in construction of **G** matrices. In general, region size of 100 SNPs led to highest prediction accuracies using BayesAS, and wGBLUP outperformed GBLUP at this region size. Our results suggest that when genetic architectures of traits favor Bayesian methods, the accuracy of multi-trait GBLUP can be as high as the Bayesian method if SNPs are weighted by the Bayesian posterior (co)variances.

Genomic prediction methods mainly fall into one of the two categories *i.e.*, (i) methods assuming a variance specific to each SNP, or to a chromosomal region (Meuwissen *et al.* 2001; Sørensen *et al.* 2012; Janss 2014), and (ii) methods assuming a common variance for all SNPs (Meuwissen *et al.* 2001; de los Campos *et al.* 2013). For instance, in one extreme BayesA method it is assumed that each SNP follows a normal distribution with null mean and a locus-specific variance $\left(\beta_{j}∼N\left(0,\sigma_{\beta_{j}}^{2}\right)\right),$ while in another extreme Ridge Regression method it is assumed that all SNPs have null means and a common variance $\left(\beta_{j}∼N\left(0,\sigma_{\beta }^{2}\right)\right).$ It has been shown that this Ridge Regression can be written as a linear mixed model at individual level (taking the sum of SNP effects as additive genetic effect of an individual) with a genomic relationship matrix (Nejati-Javaremi *et al.* 1997), **G**, which is known as genomic best linear unbiased prediction (GBLUP) (Habier *et al.* 2007). The GBLUP method is popularly used for genomic evaluation in animal breeding programs, mainly due to its straightforward implementation using the existing computer softwares (Tiezzi and Maltecca 2015; Karaman *et al.* 2016). Compared to Bayesian whole genome regression methods, GBLUP has also less computational demand particularly when the number of marker covariates greatly exceeds the number of observations, which is the case in most genome-wide analysis. Moreover, many important traits in animals are complex in nature, and are controlled by a large number of small effect genes distributed across the entire genome, favoring the infinitesimal model (Meuwissen *et al.* 2001; Hayes and Goddard 2001; Hayes *et al.* 2009; Meuwissen and Goddard 2010). For some traits, the distribution of underlying genes violates the infinitesimal model assumption of large number of genes with small effect. Thus, methods assuming SNP specific or region specific (co)variances can lead to higher accuracy of prediction than GBLUP, by accounting for the relative importance of genomic regions (Meuwissen *et al.* 2001; VanRaden *et al.* 2009).

*A priori* assumption of common variance for all loci in GBLUP results in a **G** matrix that is common to all traits. However, any trait deviates from the infinitesimal model to some degree, and different traits can be controlled by different sets of genes and different chromosomal regions (Lipkin *et al.* 2008; Zhang *et al.* 2010). Hence, it needs a **G** matrix that can reflect the genetic architecture of the traits of interest for improving genomic prediction. Zhang *et al.* (2010), for the first time, reported that the estimates from Bayesian regression methods assuming a specific variance for each SNP can be implemented in GBLUP to improve genomic prediction accuracy. Computing a trait-specific genomic relationship matrix by weighting SNPs using posterior variances from BayesB method, the authors reported higher predictive abilities than traditional GBLUP method (Zhang *et al.* 2010). Following their work, many others investigated the use of various weights for GBLUP in single-trait evaluation, either weighting SNPs individually or assigning a common weight to adjacent SNPs (Su *et al.* 2014; Calus *et al.* 2014; Tiezzi and Maltecca 2015).

A great majority of the genomic selection studies (Calus and Veerkamp 2007; Legarra *et al.* 2008; Lorenzana and Bernardo 2009; Daetwyler *et al.* 2010a; Liu *et al.* 2011; Gao *et al.* 2013; Wolc *et al.* 2013; Su *et al.* 2014) has focused on single-trait breeding value estimation. However, multi-trait methods are expected to yield more accurate predictions than single-trait methods as in traditional BLUP. Simulations have shown that multi-trait genomic prediction can lead to a considerable increase in genomic prediction accuracy (Calus and Veerkamp 2011; Jia and Jannink 2012; Guo *et al.* 2014). These studies either implemented Bayesian methods that have a higher computational demand especially for large scale of data, or used GBLUP with a **G** matrix that is common to all traits. An alternative estimation procedure can be to combine important features of both methods such that any deviation from the infinitesimal model are accounted for without compromising the simplicity in application. In a bivariate model, for instance, such a procedure should account for heterogeneous covariances between SNP effects on two traits as well as heterogeneous variances of SNP effects on each trait. In the framework of bivariate GBLUP, this immediately requires that genomic relationship matrices are able to account for heterogeneous variances and and covariances accross the genome.

The aim of this study is to (i) extend the weighted single-trait GBLUP methodology to multi-trait case, (ii) compare the performance of proposed methodology with unweighted single and multi-trait GBLUP methods, and (iii) investigate the effect of two weighting strategies (*i.e.*, single or group SNP weighting).

## Material and Methods

### Data Sets

Five generations of genotype data were simulated based on 50K haplotype data of 2,200 animals from Danish Holstein population. Only the SNPs (11,154) on first five chromosomes were used. A fixed ratio of male:female, 1:10, was assumed throughout the simulated generations. Thus, at each generation, 200 and 2,000 animals were assumed to be males and females, respectively. Each male was randomly mated with 10 females, and one mating per sire was replicated so as to retain the population size at 2,200 for each generation. The number of recombinations on each chromosome was determined using a random variable drawn from a Poisson distribution, under the assumption that the length of a chromosome in the Morgan’s unit in the linkage map is the lambda parameter (Yamamoto *et al.* 2016). Mutation was not considered in the simulations. Animals in the base population (hereinafter referred to as G0) were used to form a reference population, while animals in the simulated generations (hereinafter referred to as G1-G5) were used to form the test populations.

Some SNPs were assigned to QTL in the following way. Each chromosome was divided into windows of 1 million bp, yielding 159, 139, 127, 121 and 125 bins for chromosomes 1-5, respectively. Then, a bin was randomly selected, and a random number, $r_{w},$ was drawn from a uniform distribution U(0,0.15). A SNP was randomly selected and designated as QTL among those SNPs, if any, which met the condition of $0.15-r_{w}<{\text{MAF}}_{j}\leq 0.15+r_{w},$ where ${\text{MAF}}_{j}$ is the minor allele frequency of the SNP. Each bin was allowed to include one QTL at maximum. The restriction in MAF for QTL was based on the assumption that QTL in general have relatively low MAF. Two traits were considered, and total number of QTL was set at 200 or 500, which were excluded from the final data set of SNP. Averaged over the replicates, MAFs of the QTL and SNPs were 0.15 and 0.26, respectively.

All QTL were assigned into three groups according to their causal relationships with the traits. This was done by assuming a percentage of the total QTL (82%) had pleiotropic effects on two traits, while the rest of the QTL had effects on either of the traits. Two correlated gamma variables (*i.e.*, QTL effects) with marginal distributions of G(0.4, 1.66) were simulated for the pleiotropic QTL (Dvorkin 2012). It was assumed that the pleiotropic QTL had effects either on the same or on the opposite direction for the two traits. The 78% of those QTL were assigned to a correlation between effects on two traits of 0.90, and 22% of -0.90 randomly, to accomplish a genetic correlation of about 0.45. The effects of trait-specific QTL were sampled from a Gamma distribution, G(0.4,1.66), and were assigned a positive or negative sign at random.

Phenotypic values of the two traits were simulated to have heritabilities of 0.1 and 0.4, which represents low (*L*) and high (*H*) heritability traits, respectively. The phenotypic values of individual *i* on two traits, $y_{Li}$ and $y_{Hi},$ adjusted for fixed effects can be given as:

$$\begin{array}{c} y_{Li}=u_{Li}+e_{Li}\\ =q_{1,i}^{\prime }\alpha_{1}+q_{2,i}^{\prime }\alpha_{2,L}+e_{Li}\\ y_{Hi}=u_{Hi}+e_{Hi}\\ =q_{2,i}^{\prime }\alpha_{2,H}+q_{3,i}^{\prime }\alpha_{3}+e_{Hi} \end{array}$$

where $u_{Li}$ and $u_{Hi}$ are the breeding values of individual *i* for traits *L* and *H*, respectively. $q_{1,i}$ and $q_{3,i}$ are the vectors of the genotypes of QTL that determine either trait *L* or *H*, and $q_{2,i}$ is the genotype vector of QTL with effect on both traits, for individual *i*. The $\alpha_{1},\alpha_{2}$s and $\alpha_{3}$ are vectors of QTL effects for the corresponding three groups of QTL, and $e_{Li}$ and $e_{Hi}$ are the random residual effects. Random residual effects were sampled from $N\left(0,\left[\begin{array}{cc} I\sigma_{e_{L}}^{2} & 0\\ 0 & I\sigma_{e_{H}}^{2} \end{array}\right]\right),$ where the sizes of $\sigma_{e_{L}}^{2}$ and $\sigma_{e_{H}}^{2}$ were determined according to heritabilities of 0.1 and 0.4, respectively. All animals had genotypes and phenotypes on both traits. In total, 10 replicates were generated. Averaged over the replicates, the heritabilities for traits *L* and *H* were 0.1 and 0.4, respectively, and the genetic correlation between the traits was 0.47.

### Models and Methods

#### Definition of a basic multi-trait model:

In practice, QTL are unobserved, and breeding value estimates are based on markers instead of QTL. A multi-trait model with marker effects can be written as

y=Xβ+e (1)

where y is the vector of phenotypes corrected for the effects other than genetic effect, X is the matrix of genotypes (centered) for *k* markers, β is the vector of marker effects, and e is the vector of random residual effects. Equation (1) can be explicitly written as follows.

$$\left[\begin{array}{c} y_{L}\\ y_{H} \end{array}\right]=\left[\begin{array}{cc} X_{L} & 0\\ 0 & X_{H} \end{array}\right]\left[\begin{array}{c} \beta_{L}\\ \beta_{H} \end{array}\right]+\left[\begin{array}{c} e_{L}\\ e_{H} \end{array}\right]$$

In the case that two traits are measured on the same animals, marker genotype matrices $X_{L}=X_{H},$ and these matrices will be denoted as $X_{0}$ hereinafter, to simplify the demonstration. A typical assumption for residuals, $e\prime =\left[e_{L}^{\prime },e_{H}^{\prime }\right],$ is $e|R_{0}∼N\left(0,R_{0}⊗I_{n}\right),$ where $R_{0}=\left[\begin{array}{cc} \sigma_{e_{L}}^{2} & \sigma_{e_{LH}}\\ \sigma_{e_{LH}} & \sigma_{e_{H}}^{2} \end{array}\right].$. Assuming all the genetic variance is captured by markers, vectors of breeding values of the animals for traits *L* and *H* are $u_{L}=X_{0}\beta_{L}$ and $u_{H}=X_{0}\beta_{H},$ respectively.

#### Multi-trait genomic BLUP:

We will first start with a multi-trait BLUP model assuming independence of SNP effects on different loci, and homogeneous SNP (co)variances across the genome. We will then make a connection between that model and the traditional BLUP model based on genomic relationship matrix (GBLUP). Finally, we will relax the assumption of homogeneous SNP (co)variances, and introduce a multi-trait GBLUP model where SNP (co)variances across the genome are assumed to be heterogeneous (wGBLUP).

When constant (co)variances are assumed for all the SNPs, $\beta |B_{0}∼N\left(0,B_{0}⊗I_{k}\right),$ where $B_{0}=\left[\begin{array}{cc} \sigma_{\beta_{L}}^{2} & \sigma_{\beta_{LH}}\\ \sigma_{\beta_{LH}} & \sigma_{\beta_{H}}^{2} \end{array}\right].$. BLUP of breeding values, $u\prime =\left[u_{L}^{\prime },u_{H}^{\prime }\right],$ are

$$\begin{array}{c} \hat{u}=\mathrm{Cov}\left(u,y\prime \right)\mathrm{Var}{\left(y\right)}^{-1}y\\ =\mathrm{Cov}\left(X\beta ,y\prime \right)\mathrm{Var}{\left(y\right)}^{-1}y\\ =XBX\prime {\left[XBX\prime +R\right]}^{-1}y\\ ={\left[I_{2n}+{\left(XBX\prime \right)}^{-1}R\right]}^{-1}y \end{array}$$ (2)

where $XBX\prime =\left(\begin{array}{cc} X_{0}\sigma_{\beta_{L}}^{2}X_{0}^{\prime } & X_{0}\sigma_{\beta_{LH}}X_{0}^{\prime }\\ X_{0}\sigma_{\beta_{LH}}X_{0}^{\prime } & X_{0}\sigma_{\beta_{H}}^{2}X_{0}^{\prime } \end{array}\right)$ and $R=R_{0}⊗I_{n}.$ Assuming Hardy-Weinberg equilibrium, and homogeneous (co)variance for SNPs, total genetic variance and covariances are (Morota and Gianola 2014) $\sigma_{u_{L}}^{2}=\sum 2p_{j}q_{j}\sigma_{\beta_{L}}^{2},$ $\sigma_{u_{H}}^{2}=\sum 2p_{j}q_{j}\sigma_{\beta_{H}}^{2}$ and $\sigma_{u_{LH}}=\sum 2p_{j}q_{j}\sigma_{\beta_{LH}},$ respectively, where $p_{j}$ and $q_{j}$ are the allele frequencies of 2^nd^ and 1^st^ alleles at loci *j*. Using the fact that

$$\sigma_{\beta_{L}}^{2}=\frac{\sigma_{u_{L}}^{2}}{\sum 2p_{j}q_{j}},\;\sigma_{\beta_{H}}^{2}=\frac{\sigma_{u_{H}}^{2}}{\sum 2p_{j}q_{j}},\;\sigma_{\beta_{LH}}=\frac{\sigma_{u_{LH}}}{\sum 2p_{j}q_{j}},$$

and in turn replacing respective parameters in equation (2), we get

$$\begin{array}{c} \hat{u}={\left[I_{2n}+{\left(\begin{array}{cc} X_{0}\sigma_{\beta_{L}}^{2}X_{0}^{\prime } & X_{0}\sigma_{\beta_{LH}}X_{0}^{\prime }\\ X_{0}\sigma_{\beta_{LH}}X_{0}^{\prime } & X_{0}\sigma_{\beta_{H}}^{2}X_{0}^{\prime } \end{array}\right)}^{-1}R\right]}^{-1}y\\ ={\left[I_{2n}+{\left(\begin{array}{cc} \sigma_{u_{L}}^{2}X_{0}DX_{0}^{\prime } & \sigma_{u_{LH}}X_{0}DX_{0}^{\prime }\\ \sigma_{u_{LH}}X_{0}DX_{0}^{\prime } & \sigma_{u_{H}}^{2}X_{0}DX_{0}^{\prime } \end{array}\right)}^{-1}R\right]}^{-1}y\\ ={\left[I_{2n}+{\left(\begin{array}{cc} \sigma_{u_{L}}^{2}G & \sigma_{u_{LH}}G\\ \sigma_{u_{LH}}G & \sigma_{u_{H}}^{2}G \end{array}\right)}^{-1}R\right]}^{-1}y \end{array}$$

where $G=X_{0}DX_{0}^{\prime },$ and **D** is a diagonal matrix with $d_{jj}=\frac{1}{\sum 2p_{j}q_{j}}$ (VanRaden 2008). Hence, only a single relationship matrix needs to be computed, and

$$\left[\begin{array}{c} u_{L}\\ u_{H} \end{array}\right]∼N\left[\begin{array}{cc} 0, & \left(\begin{array}{cc} \sigma_{u_{L}}^{2}G & \sigma_{u_{LH}}G\\ \sigma_{u_{LH}}G & \sigma_{u_{H}}^{2}G \end{array}\right) \end{array}\right].$$

Hereinafter, this multi-trait BLUP model using a single relationship matrix will be referred to as multi-trait GBLUP.

Assume that each SNP has a specific (co)variance, and that β|B∼N(0,B), with $B=\left[\begin{array}{cc} B_{L} & B_{LH}\\ B_{LH} & B_{H} \end{array}\right].$. Here, $B_{L}$ and $B_{H}$ are diagonal matrices of SNP variances for traits *L* and *H*, respectively, and $B_{LH}$ is a diagonal matrix of the covariances for the SNP effects on the traits. BLUP of breeding values are

$$\begin{array}{c} \hat{u}={\left[I_{2n}+{\left(\begin{array}{cc} X_{0}B_{L}X_{0}^{\prime } & X_{0}B_{LH}X_{0}^{\prime }\\ X_{0}B_{LH}X_{0}^{\prime } & X_{0}B_{H}X_{0}^{\prime } \end{array}\right)}^{-1}R\right]}^{-1}y\\ ={\left[I_{2n}+{\left(\begin{array}{cc} \sigma_{u_{L}}^{2}X_{0}D_{L}X_{0}^{\prime } & \sigma_{u_{LH}}X_{0}D_{LH}X_{0}^{\prime }\\ \sigma_{u_{LH}}X_{0}D_{LH}X_{0}^{\prime } & \sigma_{u_{H}}^{2}X_{0}D_{H}X_{0}^{\prime } \end{array}\right)}^{-1}R\right]}^{-1}y\\ ={\left[I_{2n}+{\left(\begin{array}{cc} \sigma_{u_{L}}^{2}G_{L} & \sigma_{u_{LH}}G_{LH}\\ \sigma_{u_{LH}}G_{LH} & \sigma_{u_{H}}^{2}G_{H} \end{array}\right)}^{-1}R\right]}^{-1}y \end{array}$$

with

$$d_{L,j}=\frac{\sigma_{\beta_{L,j}}^{2}/\sigma_{\beta_{L}}^{2}}{\sum 2p_{j}q_{j}},d_{H,j}=\frac{\sigma_{\beta_{H,j}}^{2}/\sigma_{\beta_{H}}^{2}}{\sum 2p_{j}q_{j}},d_{LH,j}=\frac{\sigma_{\beta_{LH,j}}/\sigma_{\beta_{LH}}}{\sum 2p_{j}q_{j}},$$

where $d_{L,j},d_{H,j}$ and $d_{LH,j}$ are entries for the *j*’th diagonal of corresponding D matrices, $\sigma_{\beta_{L,j}}^{2}$ and $\sigma_{\beta_{H,j}}^{2}$ are the SNP variances for traits *L and H*, respectively, and $\sigma_{\beta_{LH,j}}$ is the covariance of the *j*th SNP between traits. It is more convenient to write

$$\left[\begin{array}{c} u_{L}\\ u_{H} \end{array}\right]∼N\left[\begin{array}{cc} 0, & \left(\begin{array}{cc} \sigma_{u_{L}}^{2}G_{L} & \sigma_{u_{LH}}G_{LH}\\ \sigma_{u_{HL}}G_{LH} & \sigma_{u_{H}}^{2}G_{H} \end{array}\right) \end{array}\right]$$

where $G_{L}$ and $G_{H}$ are the genomic relationship matrices that account for the heterogeneous variances, while $G_{LH}$ is the genomic relationship matrix that accounts for heterogeneous covariances across the genome. Hereinafter multi-trait BLUP model based on these genomic relationship matrices will be referred to as multi-trait weighted GBLUP (wGBLUP).

We also used single-trait weighted and unweighted GBLUP to predict breeding values in this study. In the single-trait analysis, the distribution of breeding values were assumed to follow $u_{L}∼N\left(0,\sigma_{u_{L}}^{2}G_{L}\right)$ and $u_{H}∼N\left(0,\sigma_{u_{H}}^{2}G_{H}\right)$ in weighted GBLUP, and $u_{L}∼N\left(0,\sigma_{u_{L}}^{2}G\right)$ and $u_{H}∼N\left(0,\sigma_{u_{H}}^{2}G\right)$ in unweighted GBLUP.

#### Multi-trait BayesAS:

The multi-trait BayesAS method is an extension of the method proposed by Janss (2014). The assumption in this method is that SNPs in a genomic region have the same genomic (co)variance, but SNPs in different genomic regions have different genomic (co)variances (Li *et al.* 2017). The model can be written as

$$y_{t}=\mu_{t}+\sum_{s=1}^{n_{region}}X_{s}\beta_{ts}+e_{t}\left(t=L,H\right)$$

where $y_{t}$ is the vector of phenotypes, $\mu_{t}$ is the overall mean, $X_{s}$ is the matrix of marker genotypes for region *s*, $\beta_{ts}$ is the vector of marker effects of region *s* for trait *t*, $e_{t}$ is the vector of random residual effects. For the region *s*, SNP effects across traits are correlated and are formulated by the following hierarchical model

$$\beta_{ts}=r_{t}s_{0,s}+r_{ts}s_{1,s}+\beta_{ts}^{*}$$ (3)

In the hierarchical model, a SNP effect is decomposed to three components. The vectors $s_{0,s}$ and $s_{1,s}$ are subsets of latent variables $s_{0}$ and $s_{1},$ respectively, and are common to all traits in the analysis. The vectors $s_{0}$ and $s_{1}$ are vectors with SNP effects transformed to an eigen-vector space (Janss 2014). The purpose of the latent vectors $s_{0}$ and $s_{1}$ is to model covariances between traits. Regression coefficients $r_{t}$ and $r_{ts}$ determine the sizes of the covariances. Hence, average covariance between SNP effects are modeled through $r_{t}s_{0,s},$ while the deviation of region *s* from this common covariance is modeled through $r_{ts}s_{1,s}.$ In BayesAS, the elements of the vectors $s_{0}$ and $s_{1}$ are estimated in a hierarchical model specification, where the SNP effects on each trait are taken as a known “response”, the $r_{t}$ and $r_{ts}$ parameters are taken as known scaling parameters, and $s_{0}$ and $s_{1}$ are updated by a regular mixed model equation. Then, taking $s_{0}$ and $s_{1}$ as known, $r_{t}$ and $r_{ts}$ are updated. Vector of residual SNP effects, $\beta_{ts}^{*},$ consists of the SNP effects, which are uncorrelated across traits. For a SNP having effect only on one trait, the sum of the two regression terms $\left(r_{t}s_{0,s}+r_{ts}s_{1,s}\right)$ is expected to be null, and for a SNP having the same effect on both traits, $\beta_{ts}^{*}$ is expected to be null. We assumed following priors for the parameters of the models

$$\begin{array}{c} r_{t}∼U\left(-\infty ,\infty \right),s_{0}∼N\left(0,I\right),\\ r_{ts}∼N\left(0,\sigma_{r_{t}}^{2}\right),\sigma_{r_{t}}^{2}∼U\left(0,\infty \right),s_{1}∼N\left(0,I\right),\\ \beta_{ts}^{*}∼N\left(0,I\sigma_{\beta_{t}^{\text{*}}}^{2}\right),\sigma_{\beta_{t}^{*}}^{2}∼U\left(0,\infty \right). \end{array}$$

Variance and covariance for each SNP in each region can be computed as $\mathrm{var}\left(\beta_{ts}\right)=r_{t}^{2}+r_{ts}^{2}+\sigma_{\beta_{t}^{*}}^{2}$ and $\mathrm{cov}\left(\beta_{Ls},\beta_{Hs}\right)=r_{L}r_{H}+r_{Ls}r_{Hs}$ (Li *et al.* 2017). Single-trait BayesAS was also used, where equation (3) reduces to $\beta_{ts}=r_{ts}s_{1,s}+\beta_{ts}^{*},$ and the variances for each SNP in each region becomes $\mathrm{var}\left(\beta_{ts}\right)=r_{ts}^{2}+\sigma_{\beta_{t}^{*}}^{2}.$ Priors for single-trait BayesAS model were as defined for multi-trait BayesAS, with $\sigma_{\beta_{t}^{*}}^{2}$ set at the value from multi-trait analysis.

### Statistical Analysis

We aimed to compare the prediction accuracies of BayesAS, GBLUP and wGBLUP in single and multi-trait genomic prediction. Four different sizes of genomic regions (one SNP, 100 SNPs, one chromosome and whole genome) were considered using BayesAS. Posterior (co)variance components of the traits from BayesAS models with different region sizes were used in GBLUP, and posterior (co)variances of SNPs were used to calculate weights for wGBLUP. It is worth noting that (co)variance components of traits may vary among the BayesAS analyses with different region sizes to some extent (Li *et al.* 2017), which in turn may result in accuracies from GBLUP analysis to vary within single and multi-trait analysis.

The analysis of single and multi-trait BayesAS model was carried out using the BAYZ software (www.bayz.biz). Chain length consisted of 20,000 cycles, and the first 5,000 cycles were considered as burn-in. Thinning interval was set to 10, and in total 1,500 samples were saved for the posterior analysis. Mean value of the posterior samples was considered as the estimate of each parameter. GBLUP and wGBLUP results were obtained by solving mixed model equations in R (R Core Team 2017).

Accuracy of prediction from different methods was calculated as the correlation between true and estimated breeding values of animals in test populations. Results presented here were the average over 10 replicates.

Single and multi-trait BayesAS models assuming different region sizes were compared for each trait, separately. Prediction accuracies for all methods were compared for each trait and each scenario of region size. Those comparisons were performed by two-sided paired *t*-tests, for which accuracies were paired across each replicate for the same test population, and based on a *p*-value of 0.05 with Bonferroni correction.

### Data Availability

Supplemental material available at Figshare: https://figshare.com/s/1a1886f38d25b6f389ff. Files at this URL are, real and simulated genotypes, and File S1, which includes Tables S1 and S2 for accuracies of generations 2-5 under two QTL scenarios. The genotypes and the methodology described previously are sufficient to reproduce the results of this study. The authors affirm that all data necessary for confirming the conclusions of the article are present within the article, figures, and tables.

## Results

### Genomic prediction using BayesAS

Prediction accuracies from single and multi-trait BayesAS models for different region sizes are presented in Tables 1-2, for test animals in G1.

**Table 1:** Accuracies of genomic prediction for test animals in generation 1 using different methods with varying region sizes, for 200 QTL scenario

		Single-Trait			Multi-Trait		
Trait*^a^*	Region Size*^b^*	BayesAS	GBLUP	wGBLUP*^c^*	BayesAS	GBLUP	wGBLUP
*L**^d^*	1 SNP	_b_0.495^c^	0.493^c^	0.499^bc^	_b_0.539^a^	0.540^a^	0.535^ab^
	100 SNPs	_a_0.532^c^	0.493^d^	0.530^c^	_a_0.597^a^	0.532^c^	0.590^b^
	1 Chr	_b_0.488^b^	0.486^b^	0.498^b^	_b_0.557^a^	0.543^a^	0.558^a^
	WG	_b_0.488^b^	0.489^b^	0.482^b^	_b_0.541^a^	0.542^a^	0.542^a^
*H*	1 SNP	_b_0.714^a^	0.702^b^	0.715^a^	_bc_0.713^a^	0.705^ab^	0.714^a^
	100 SNPs	_a_0.755^a^	0.703^b^	0.757^a^	_a_0.761^a^	0.705^b^	0.760^a^
	1 Chr	_b_0.711^ab^	0.702^c^	0.711^ab^	_b_0.714^a^	0.706^bc^	0.715^a^
	WG	_c_0.701^a^	0.701^a^	0.688^a^	_c_0.705^a^	0.705^a^	0.706^a^

a *L* and *H*: low (0.1) and high (0.4) heritability traits, respectively.

b Chr: chromosome; WG: Whole genome.

c wGBLUP: weighted GBLUP.

d Different alphabets mean significantly different values at a type one error rate of 0.05 with Bonferroni correction. Subscripts and superscripts stand for comparisons within column and row, respectively, for each trait.

**Table 2:** Accuracies of genomic prediction for test animals in generation 1 using different methods with varying region sizes, for 500 QTL scenario

		Single-Trait			Multi-Trait		
Trait*^a^*	Region Size*^b^*	BayesAS	GBLUP	wGBLUP*^c^*	BayesAS	GBLUP	wGBLUP
*L**^d^*	1 SNP	_ab_0.486^b^	0.486^b^	0.484^b^	_a_0.527^a^	0.527^a^	0.527^a^
	100 SNPs	_a_0.496^cd^	0.486^d^	0.494^c^^d^	_a_0.551^a^	0.522^b^^c^	0.547^a^^b^
	1 Chr	_bc_0.475^b^	0.484^b^	0.485^a^^b^	_a_0.527^a^	0.528^a^	0.528^a^
	WG	_c_0.473^b^	0.474^b^	0.475^b^	_a_0.529^a^	0.528^a^	0.528^a^
*H*	1 SNP	_b_0.701^a^	0.696^c^	0.701^a^^b^^c^	_b_0.701^a^	0.699^b^^c^	0.701^a^
	100 SNPs	_a_0.724^a^	0.696^c^	0.723^a^	_a_0.727^a^	0.699^b^	0.725^a^
	1 Chr	_bc_0.698^cd^	0.696^b^^d^	0.697^a^^b^^c^^d^	_b_0.702^abcd^	0.699^a^^c^	0.703^a^^b^
	WG	_c_0.695^c^	0.695^c^	0.684^c^	_b_0.700^ab^	0.699^b^^c^	0.700^a^

^a^ *L* and *H*: low (0.1) and high (0.4) heritability traits, respectively.

^b^ Chr: chromosome; WG: Whole genome.

^c^ wGBLUP: weighted GBLUP.

^d^ Different alphabets mean significantly different values at a type one error rate of 0.05 with Bonferroni correction. Subscripts and superscripts stand for comparisons within column and row, respectively, for each trait.

Accuracies from multi-trait analysis were significantly higher than their single-trait counterparts regardless of the region size or the number of QTL, for trait *L*. For trait *H*, on the other hand, accuracies from single and multi-trait analyses were similar at all region sizes, except for the region size of whole genome in 500 QTL scenario.

Region size of 100 SNPs resulted in the highest accuracies in single and multi-trait analyses, for both traits. In the scenario of 500 QTL, accuracy from region size of 100 SNPs was not significantly higher than that from region size of 1 SNP, but than from one chromosome or whole genome, for trait *L* using a single-trait model. For this trait, accuracy for the region size of 100 SNPs was more than 2 percentage points higher than those for other region sizes using a multi-trait model, however, it was not significant.

### Weighted GBLUP using SNP (co)variances From BayesAS

After fitting BayesAS models with different region sizes, posterior (co)variance components of the traits from these models were used in GBLUP, and posterior (co)variances of SNPs were used to calculate weights for wGBLUP. Accuracies from GBLUP and wGBLUP methods are presented in Tables 1 and 2 along with the accuracies from BayesAS methods, for test animals in G1. It is worth noting that what varied among the different region sizes for single or multi-trait GBLUP analyses were the estimates of (co)variance components of traits due to using BayesAS with different region sizes, while the relationship matrices remained unchanged.

Similar to BayesAS model, in general, accuracies from multi-trait wGBLUP and GBLUP analysis were significantly higher than their single-trait counterparts for trait *L*. For trait *H*, on the other hand, accuracies from single and multi-trait wGBLUP analyses were generally similar in both QTL scenarios.

In the scenario of 200 QTL, accuracies for trait *L* from wGBLUP were significantly higher than those from GBLUP, when the weighing factors were the (co)variances from BayesAS with region size of 100 SNPs, for both single and multi-trait analysis. In the scenario of 500 QTL, the accuracy for trait *L* from wGBLUP was higher than GBLUP (2.5 percentage points) using a multi-trait model with region size of 100 SNPs, however, it was not significant. There was no significant difference between wGBLUP and GBLUP, when the region was one SNP, one chromosome or whole genome for trait *L*, regardless of the number of QTL.

For trait *H*, prediction accuracies from wGBLUP models when weights were obtained from BayesAS with region sizes of 100 SNPs, were significantly higher than those from corresponding GBLUP models, regardless of the number of QTL. Weighted GBLUP was similar or superior to traditional GBLUP for region sizes of one SNP, one chromosome or whole genome, for trait *H*.

Although not always statistically significant, analysis using (co)variance estimates from BayesAS in construction of **G** matrices resulted in higher accuracies than GBLUP, when the accuracies of corresponding BayesAS model was higher than those of GBLUP. Moreover, prediction accuracies of breeding values from wGBLUP were generally as high as those from BayesAS models.

### Change in accuracy Over generations

**Figure 1:**
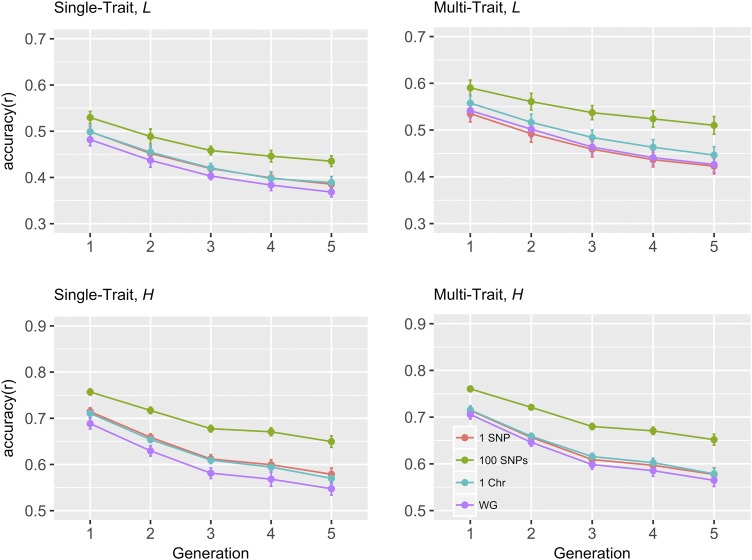
Change in prediction accuracy for weighted GBLUP over generations with varying regions sizes of genome regions, for 200 QTL scenario. *L* and *H*: low (0.1) and high (0.4) heritability traits, respectively; different colors represent different region sizes; Chr: chromosome; WG: whole genome (or equivalently, GBLUP).

**Figure 2:**
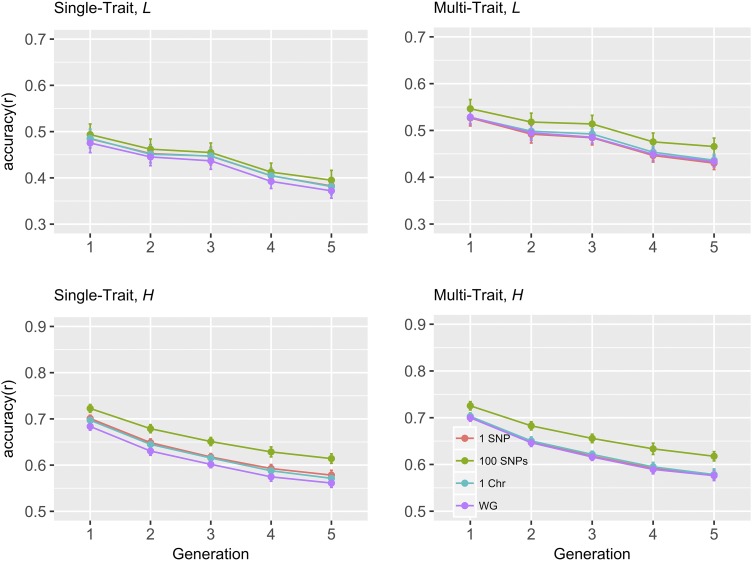
Change in prediction accuracy for weighted GBLUP over generations with varying regions sizes of genome regions, for 500 QTL scenario. *L* and *H*: low (0.1) and high (0.4) heritability traits, respectively; different colors represent different region sizes; Chr: chromosome; WG: whole genome (or equivalently, GBLUP).

Figures 1 and 2 show the prediction accuracy of breeding values using wGBLUP method for five consecutive generations, when the total number of QTL were 200 and 500, respectively. It is worth noting that wGBLUP model with region size of whole genome is consistent with GBLUP, where all SNPs are implicitly assumed to have the same (co)variance. For all four region sizes considered, accuracy decreased with generations regardless of the number of QTL. The rate of decay in accuracy for a region size of 100 SNPs was slightly lower than that for other region sizes when the total number of QTL was 200, particularly in multi-trait setting for trait *L*. When the number of QTL was 200, substantial accuracy was still retained with a region size of 100 SNPs using the multi-trait wGBLUP method, even after five generations. Accuracies from 500 QTL scenario (Figure 2) decayed slower compared to 200 QTL scenario (Figure 1), when the region size was whole genome. The accuracies of (single or multi-trait) BayesAS and wGBLUP were very similar in all five generations (Tables S1 and S2).

## Discussion

### Region size in BayesAS

A wide range of statistical methods is available for single-trait genomic prediction, which mainly differ in the assumptions on SNP effects (Meuwissen *et al.* 2001; Gianola *et al.* 2009; de los Campos *et al.* 2013). Among those methods, an extreme is the BayesA method, where prior distribution of each SNP effect is assumed to be normal with a SNP specific variance, $\sigma_{\beta_{j}}^{2}.$ Those variances are further assumed to follow a scaled inverted chi-square distribution with some scale and degree of freedom (*df*) parameters. This was criticized by Gianola *et al.* (2009) as not following Bayesian learning, because posterior SNP variances have only one additional *df* compared to their prior. This can be overcome by assigning a common variance for a subset of SNPs, and therefore, posterior distribution of each SNP variance (within a group consisting of $k_{\text{S}}$ SNPs) will have $k_{\text{S}}$ *df* in addition to prior *df* (Gianola *et al.* 2009). These subsets can be achieved by grouping SNPs based on fixed length of genomic region, or fixed number of adjacent SNPs in each subset. This is reasonable, because the SNPs in a region likely inherit together, and also likely to be in linkage disequilibrium (LD) with the same QTL (Sørensen *et al.* 2012; Gebreyesus *et al.* 2017).

Some of the existing single-trait Bayesian whole genome regression models have been extended to accommodate more than one trait at a time (Calus and Veerkamp 2011; Jia and Jannink 2012; Hayashi and Iwata 2013). An issue with those models is that when two traits are considered simultaneously, the models are restricted to two situations for a locus *i.e.*, the locus has effect on both traits, or none of them. Cheng *et al.* (2018) developed a set of models that can accommodate all possible situations, that is, in terms of two traits, their models can handle also with the situation where a locus has effect on one trait, but not on the other. With their new multi-trait BayesCΠ model (Cheng *et al.* 2018), they reported the same as or higher accuracies than the model considering two situations only (Jia and Jannink 2012), using real and simulated data sets.

The Bayesian method used in this study, BayesAS, also assumes that a locus simultaneously affects all the traits, but allows the inclusion of priors for genomic regions of any size. This way, the method exploits the genomic regions that show correlations between traits, which deviate from the genome-wide correlation (Li *et al.* 2017). Assuming a common (co)variance for a group of adjacent SNPs is not as flexible as assuming a (co)variance specific to each SNP, but it increases the power in estimation of SNP (co)variances. Taking advantage of grouping adjacent SNPs, therefore, might be critical to achieving a high prediction accuracy, by better exploiting the local deviations from the average genome-wide (co)variance.

Special cases of BayesAS method occur when the region size is one SNP or whole genome, which has similar assumptions to BayesA or GBLUP, respectively. Because our primary aim was to develop a methodology to improve accuracy of multi-trait genomic prediction using GBLUP via weighted **G** matrices, we only investigated a limited number of region sizes (one SNP, 100 SNPs, one chromosome or whole genome). Our results demonstrated the potential of improving genomic prediction accuracy by assigning priors to genomic regions for both single and multi-trait analysis via BayesAS method. Highest accuracies were reached with BayesAS when the region size was 100 SNPs, regardless of the number of QTL, however, some of those for trait *L* in 500 QTL scenario were not significantly higher than all other region sizes considered here (Tables 1 and 2).

Li *et al.* (2017) estimated genetic parameters for milk, fat and protein yields of Chinese and Nordic Holstein cattle using BayesAS model, where the same trait in these countries was treated as different traits, and analyzed using a bivariate model. They reported that region size of 100 SNPs resulted in similar estimates of variance components as region sizes of 200 or 400, but more accurate estimates than region size of 50, using a 50K SNP panel. Fitting a Bayesian multi-trait mixed model that uses latent variables to fit (co)variance structures, Sørensen *et al.* (2012) estimated genomic (co)variances for six mastitis resistance related traits in Danish cows, for some region sizes. Their results using half-overlapping region size of 100 SNPs on BTA19 indicated the existence of different (co)variance patterns for different traits. Brøndum *et al.* (2012) modified the single-trait BayesR model to allow incorporating prior information on genomic regions, and referred to it as BayesRS in an across-breed genomic prediction study for protein, fat, and milk yield. They reported that, among varying sizes of genome regions they examined, the highest accuracies were obtained with the region size of 100 SNPs. However, the SNP panel used in (Brøndum *et al.* 2012) was dense consisting of (before data editing) 777K SNPs, and therefore, the region size of 100 SNPs corresponds to a region size of roughly 5-10 SNPs in the 50K panel.

In a recent study, Gebreyesus *et al.* (2017) used the model for multi-trait genomic prediction, and reported substantial improvements in reliabilities using a region size of 100 SNPs compared to the reliabilities from a bivariate GBLUP model, for most of the milk protein composition traits in Danish Holstein cattle. The extent of LD is highly variable in different populations, and it also varies with respect to SNP density (Goddard and Hayes 2009). The decision of optimal region size is, therefore, crucial to obtain highest accuracy of genomic prediction (Gebreyesus *et al.* 2017).

### Weighted GBLUP using SNP (co)variances From BayesAS

Computation of relationship matrices by weighting SNPs was first proposed by Zhang *et al.* (2010), and higher accuracies were reported for wGBLUP than for GBLUP, using single-trait analysis. Following their work, the use of weights to compute genomic relationships has been studied extensively, and many different weights such as *p*-values from GWAS studies, genetic variance at a locus etc. were used (Su *et al.* 2014; Calus *et al.* 2014; Tiezzi and Maltecca 2015).

In the applications of weighted GBLUP, SNP specific weights were either used to weight SNPs individually, or averaged (or summed) over adjacent SNPs to assign a common weight to a set of SNPs (Su *et al.* 2014; Calus *et al.* 2014; Zhang *et al.* 2016). Su *et al.* (2014) reported that using the mean variance of 30-SNP window as weights in GBLUP improved the reliabilities up to one percent for four production traits and mastitis, compared with single SNP weighting. Zhang *et al.* (2016) used an iterative weighting procedure, where the SNP variances estimated in the previous iteration were used to weight SNPs in the current iteration when building a **G** matrix for single-step GBLUP (Christensen and Lund 2010; Aguilar *et al.* 2010). Using a common weight for 20-SNP window that sums or averages the individual SNP variances improved the accuracy of prediction.

An alternative, and arguably a more straightforward approach is to assume a variance common to all SNPs in a genomic region using a Bayesian method, and then using those variances as weights to compute the **G** matrix for genomic prediction with GBLUP. The Bayesian whole genome regression method used here, BayesAS, allows assigning prior distributions for genomic regions including any number of SNPs (Li *et al.* 2017), and provides (co)variances for those regions which can directly be used to weight SNPs when building the genomic relationship matrices for multi-trait genomic prediction. The gain from a weighted GBLUP analysis depends on whether the Bayesian method used to derive weights is superior to GBLUP for traits under investigation. As discussed previously, this is highly related to the the region size when using BayesAS.

Predictions from single or multi-trait wGBLUP were not found to be superior to their GBLUP counterparts with some exceptions for high heritability trait, when each SNP assumed to have its own (co)variance using BayesAS (Tables 1 and 2). Taking full advantage of the Bayesian whole genome regression methods is much more difficult for low heritability traits, because the power to detect the right SNPs associated with the QTL is weak for low heritability trait. When weights were obtained from BayesAS with a region size of 100 SNPs, accuracies for single and multi-trait wGBLUP models were significantly higher than those for the corresponding GBLUP models, except for trait *L* when the number of QTL was 500. In fact, it is expected that there will be no advantage for Bayesian whole genome regression, and therefore, for wGBLUP over GBLUP if the simulated QTL more closely fit an infinitesimal model, unless the data are large enough (Hayes *et al.* 2009; Karaman *et al.* 2016; Cheng *et al.* 2018). In general, taking one chromosome or whole genome as one region did not yield significantly higher prediction accuracies for wGBLUP than those for GBLUP. This is due to the fact that, as region size is larger than an optimum level, the advantage of Bayesian methods starts to diminish because the assumption on (co)variance approaches to that in GBLUP.

The accuracies for wGBLUP were higher up to 4.3 percentage points for 200 QTL scenario than for 500 QTL scenario, when the region size was 100 SNPs. On the other hand, the accuracies of predictions using GBLUP showed little difference between the QTL scenarios. This is mainly due to the fact that GBLUP rely on average relationships among animals across the genome, and is less sensitive to the genetic architecture of traits (Daetwyler *et al.* 2010b; Tiezzi and Maltecca 2015).

Bayesian whole genome regression methods do not include genomic relationship matrices (*i.e.*, $G_{L},$ $G_{H}$ and $G_{LH}$) explicitly, however, these “weighted” genomic relationship matrices can be implicitly estimated during the MCMC procedure (Fernando and Gianola 2018). In multi-trait BayesA, all loci contribute to the genomic relationships, while in multi-trait BayesB, only some proportions of the total loci contribute to the genomic relationships in each cycle (Fernando and Gianola 2018). Generally speaking, under some assumptions, any Bayesian alphabet method can lead to its equivalent weighted GBLUP counterpart. Therefore, one would obtain similar accuracies for BayesB and wGBLUP, if the SNP (co)variances from BayesB were used to weight SNPs when constructing genomic relationship matrices.

In almost all cases, GBLUP was able to reach the accuracy as high as that of BayesAS models, by using posterior SNP (co)variances from the Bayesian models as weights to build **G** matrices (wGBLUP), in both single-and multi-trait analysis. This is in line with published results for single-trait weighted GBLUP (Su *et al.* 2014; Calus *et al.* 2014). To our knowledge, multi-trait weighted GBLUP has not been reported so far, although some studies constructed **G** matrices in a similar manner for across breed prediction (Zhou *et al.* 2014; Veroneze *et al.* 2016).

### Implementation of multi-trait weighted GBLUP in genomic prediction

Most recent applications of genomic prediction are based on a single-trait genomic model. Many important traits in animal breeding, however, have genetic correlations of varying size with one or more traits, and analysis of such genetically correlated traits jointly using a multi-trait model may yield more accurate predictions of breeding values compared to separate single-trait analysis. Further implementation of genomic selection in breeding programs, therefore, will require extension of current single-trait genomic evaluation models to their multi-trait versions (Janss 2014). This is straightforward for GBLUP as it requires simply replacing the numerator relationship matrix **A** in the traditional multi-trait BLUP models by the genomic relationship matrix **G** (Nejati-Javaremi *et al.* 1997; VanRaden 2008; Hayes *et al.* 2009). However, it is implicitly assumed in the commonly used **G** matrices that each loci equally contribute to the genomic (co)variance (Zhang *et al.* 2010; Janss 2014). In the situation of two correlated traits, this assumption of genetic architecture is violated if some genomic regions explain a large proportion of the total genomic variance and/or covariance, while other regions explain only a small amount (Sørensen *et al.* 2012; Brøndum *et al.* 2012; Li *et al.* 2017; Gebreyesus *et al.* 2017).

Bayesian whole genome regression methods can be used to relax the assumption of equal contribution of each loci to the genomic (co)variance, however, they have computational disadvantages which make them difficult to be used in routine genomic evaluations. Weighted GBLUP can make it possible to avoid computational costs of Bayesian methods, while maintaining a higher prediction accuracy compared to traditional GBLUP. Such a procedure still requires to perform Bayesian analysis to obtain weights, but less frequently compared to the number of evaluations. Su *et al.* (2014) reported that weights obtained from a data set having a lag up to three years did not reduce the reliability of genomic prediction in the evaluation of a dairy cattle population.

In practical genomic evaluations, variance components are generally estimated using a linear mixed model at individual level via REML or Bayesian methods (Hayes *et al.* 2009), without accounting for heterogeneous SNP (co)variance structure across the genome. Hence, BayesAS and wGBLUP models in which all SNPs are assumed to have a common (co)variance, is consistent with GBLUP. The decrease in accuracy over the five generations was larger for GBLUP than for wGBLUP with a region size of 100 SNPs (”WG” *vs.* ”100 SNPs” in Figures 1 and 2). For the region sizes considered, accuracies decreased with generations for all methods (Tables S1 and S2), in agreement with other studies (Habier *et al.* 2007; Wolc *et al.* 2011). It is worth noting that the decrease of accuracy with generations is not contradictory with the results in Su *et al.* (2014), which used same weights for three years. First, time interval of three years in a dairy cattle breeding scheme corresponds approximately to less than one generation in our simulations. Second, although the same weights were used in Su *et al.* (2014) for three years, the genomic predictions were performed using an updated phenotypic data, while not only the same weights, but also the same phenotypes from G0 were used throughout the generations in our study.

SNPs fitted in genomic prediction models not only capture the information on LD between SNPs and QTL, but also capture within-family information (Habier *et al.* 2007, 2010; Wientjes *et al.* 2013). Any change on these factors, therefore, affects the accuracy of genomic prediction. Bayesian whole genome regression methods rely more on population LD, while GBLUP relies more on within-family LD. As test population gets farther from reference population, the genetic relationships between reference and test populations become weak, and therefore, the persistence of population LD across generations becomes more important to maintain the accuracy of prediction (Habier *et al.* 2007, 2010; Tiezzi and Maltecca 2015). Due to intensive genomic selection in many countries, such as the Nordic countries, the distance between selection candidates and genomic reference population has increased. It is, therefore, very likely that most genotyped candidates will have no sire with daughter information in the reference population, at the time of they are being selected (Gao *et al.* 2013). Thus, it is important for practical applications if wGBLUP performs better than traditional GBLUP when the selection candidates are a few generations apart from the reference population.

The gain of accuracy from a multi-trait over single-trait genomic prediction is more profound for low heritability traits that are genetically correlated with a high heritability trait, and for traits which can not be measured for all individuals (Jia and Jannink 2012; Guo *et al.* 2014). When the total number of QTL was increased from 200 to 500, the advantage wGBLUP, over traditional GBLUP decreased for trait *L*. The improvement in accuracy of a low heritability trait could be similar to 500 QTL scenario, which resembles the situation of complex traits, if the methodology presented here is used in practical applications.

Due to the reduction in genotyping costs, and the interest in exchange of data among countries, reference populations now include several thousands of genotyped bulls and cows even for small breeds (Su *et al.* 2016). The reference population used here consists of a relatively small number of individuals of 2,200, which may have an impact on comparison of the models, particularly when the simulated QTL more closely fit an infinitesimal model. In order to investigate the potential usefulness of our approach, we run additional analysis combining G0 and G1 to form a reference, and G2 as test population, respectively (Table 3). Although adding more ancestral generations to the reference population is not the same as a reference population consisting of a recent generation with more animals, it can be an alternative strategy (Weng *et al.* 2016) to investigate the impact of reference population size on comparison between models. The results of BayesAS models (Table 3) demonstrate that prediction accuracies from region size of 100 SNPs was superior to those from other region sizes in both single and multi-trait analysis. More importantly, the improvement of wGBLUP over traditional GBLUP at the region size of 100 SNPs was larger than that in a smaller reference population size (Table 2), when trait *L* was controlled by relatively a large number of QTL. This suggest that weighting SNPs by posterior (co)variance estimates from Bayesian models can be a useful strategy for improving the accuracy of GBLUP, provided that the data used to derive weights is sufficiently large.

**Table 3:** Accuracies of genomic prediction for test animals in generation 2 using different methods with varying region sizes for 500 QTL scenario, when generations 0 and 1 were used as training population

		Single-Trait			Multi-Trait		
Trait*^a^*	Region Size*^b^*	BayesAS	GBLUP	wGBLUP*^c^*	BayesAS	GBLUP	wGBLUP
*L**^d^*	1 SNP	_b_0.579^b^	0.577^b^	0.578^b^	_b_0.626^a^	0.626^a^	0.627^a^
	100 SNPs	_a_0.601^b^	0.577^c^	0.599^b^	_a_0.666^a^	0.623^b^	0.661^a^
	1 Chr	_b_0.569^b^	0.577^b^	0.575^b^	_b_0.630^a^	0.627^a^	0.631^a^
	WG	_b_0.568^b^	0.570^b^	0.567^b^	_b_0.627^a^	0.627^a^	0.627^a^
*H*	1 SNP	_b_0.789^ab^	0.778^d^	0.790^a^	_b_0.786^b^	0.780^c^	0.787^ab^
	100 SNPs	_a_0.805^b^	0.778^d^	0.804^b^	_a_0.809^a^	0.780^c^	0.808^a^
	1 Chr	_c_0.779^cd^	0.778^bd^	0.778^abcd^	_c_0.782^abcd^	0.780^ac^	0.783^ab^
	WG	_c_0.779^ab^	0.778^b^	0.766^b^	_c_0.781^a^	0.780^a^	0.781^a^

^a^ *L* and *H*: low (0.1) and high (0.4) heritability traits, respectively.

^b^ Chr: chromosome; WG: Whole genome.

^c^ wGBLUP: weighted GBLUP.

^d^ Different alphabets mean significantly different values at a type one error rate of 0.05 with Bonferroni correction. Subscripts and superscripts stand for comparisons within column and row, respectively, for each trait.

### CONCLUSIONS

In this study, we aimed to develop a strategy to improve the accuracy of GBLUP in multi-trait genomic prediction. Our two step strategy involves the estimation of SNP (co)variances via a Bayesian method, and subsequent use of those (co)variances as weights in multi-trait weighted GBLUP analysis. We used BayesAS method to obtain those weights, however, other Bayesian whole genome regression methods could also be used. In general, assuming a common (co)variance for 100 adjacent SNPs led to highest prediction accuracies using BayesAS. When 100 adjacent SNPs were assigned a common weight obtained from posterior (co)variances of BayesAS, wGBLUP outperformed the traditional GBLUP. Our results demonstrate that multi-trait GBLUP can yield accuracies of genomic prediction as high as Bayesian multi-trait genomic prediction method, when SNPs are weighted by posterior SNP (co)variances from the Bayesian method. The gain from a multi-trait weighted GBLUP analysis depends on factors, such as the model used to obtain weights (*i.e.*, SNP (co)variances), genetic architecture of the traits (*e.g.*, number of QTL, traits’ heritabilities), and reference population size.
